# Ensemble Place Codes in Hippocampus: CA1, CA3, and Dentate Gyrus Place Cells Have Multiple Place Fields in Large Environments

**DOI:** 10.1371/journal.pone.0022349

**Published:** 2011-07-15

**Authors:** EunHye Park, Dino Dvorak, André A. Fenton

**Affiliations:** 1 Department of Physiology and Pharmacology, The State University of New York (SUNY), Downstate Medical Center, Brooklyn, New York, United States of America; 2 Joint Program in Biomedical Engineering SUNY, Downstate Medical Center/NYU Poly, Brooklyn, New York, United States of America; 3 Robert F. Furchgott Center for Neuroscience, The State University of New York (SUNY), Downstate Medical Center, Brooklyn, New York, United States of America; 4 Center for Neural Science, New York University, New York, New York, United States of America; University of Alberta, Canada

## Abstract

Previously we reported that the hippocampus place code must be an ensemble code because place cells in the CA1 region of hippocampus have multiple place fields in a more natural, larger-than-standard enclosure with stairs that permitted movements in 3-D. Here, we further investigated the nature of hippocampal place codes by characterizing the spatial firing properties of place cells in the CA1, CA3, and dentate gyrus (DG) hippocampal subdivisions as rats foraged in a standard 76-cm cylinder as well as a larger-than-standard box (1.8 m×1.4 m) that did not have stairs or any internal structure to permit movements in 3-D. The rats were trained to forage continuously for 1 hour using computer-controlled food delivery. We confirmed that most place cells have single place fields in the standard cylinder and that the positional firing pattern remapped between the cylinder and the large enclosure. Importantly, place cells in the CA1, CA3 and DG areas all characteristically had multiple place fields that were irregularly spaced, as we had reported previously for CA1. We conclude that multiple place fields are a fundamental characteristic of hippocampal place cells that simplifies to a single field in sufficiently small spaces. An ensemble place code is compatible with these observations, which contradict any dedicated coding scheme.

## Introduction

The discharge of the principal cells in hippocampus subregions CA1, CA3, and dentate gyrus (DG) all have the remarkable property of location-specificity, which has motivated their intense study in the effort to understand how space and memories are represented in the mammalian brain [1], [2], [3], [4], [5]. The spatial discharge properties of these ‘place cells’ have been well characterized in standard laboratory environments, which typically have a maximum linear dimension less than 1 m. In such environments, most place cells discharge action potentials in a single location called the cell's place field. Recently we compared CA1 place cell discharge in a 68-cm diameter cylinder and a larger, more natural environment, a 1.5 m×1.4 m chamber with stairs along three walls to permit movements through the space in three dimensions. We reported that dorsal CA1 place cells have single firing fields in the smaller environment but multiple, irregularly-arranged place fields in the larger chamber [6]. According to that work, the CA1 place code is fundamentally similar in small and large environments, but appeared to differ in the two environments because the available space for characterizing place cell discharge was limited in the small environment. This view is consistent with the observation that both place cells in the dentate gyrus [2], [3] and grid cells in the entorhinal cortex also have multiple firing fields [7], [8] even in standard small environments. However, the notion that place cells fundamentally have multiple firing fields contrasts with a recent report on the spatial discharge of place cells as rats traversed an 18-m linear track [9]. In that work, CA3 place cells tended to have a single place field, and the size of the field expanded with the ventral location of the cell.

**Figure 1:**
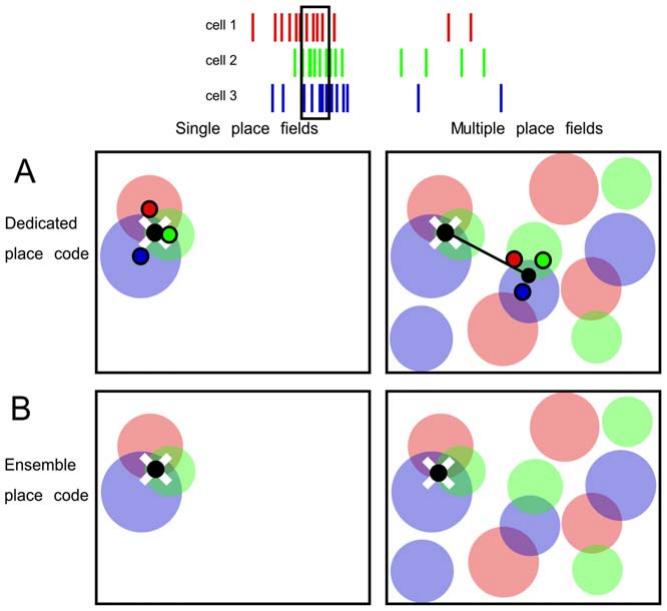
Spatial representation with dedicated place coding versus ensemble place coding. Only ensemble place codes can accurately represent location if place cells have multiple firing fields, but both ensemble and dedicated place codes are accurate if place cells have single place fields. The schematics represent a moment when three place cells discharge. Artificial rasters depict discharge of three color-coded place cells and the moment (black rectangle) that position is being estimated. The place fields of the three cells are depicted along with the subject's current location (white X), the estimate of the location (black dot), and the error of the estimate (black line). A) Given a dedicated place code, averaging the independent location estimates from each cell (corresponding-colored non-transparent dots) provides an accurate estimate of the subject's location if each cell has only one place field (left), in contrast, the location estimate is typically inaccurate if the cells have multiple place fields (right). In this depiction, each independent location estimate is the centroid of the cell's place fields. B) Given an ensemble place code, the across-cell activity pattern represents location and such place codes can work equally well if the cells have single or multiple place fields [6]. In the case that is depicted, all three cells tend to discharge together in just one region.

It seemed unlikely to us that the fundamental spatial firing characteristic of CA1 and dentate gyrus place cells would be to have multiple place fields, like entorhinal grid cells, and that the fundamental characteristic of CA3 cells would be to have single place fields. We believe it is unlikely that a single functional system like the hippocampus and entorhinal cortex system for representing space would use fundamentally different neural coding schemes. Importantly, the basic models of how the brain accurately represents the animal's current location are constrained by the correctness of these descriptions and it appears that a commonly held view remains that the fundamental firing characteristic of place cells is to have single place fields. If place cells have single place fields, then the activity of each cell is a good, independent estimate of current location and combining the independent location estimates from individual cells can provide an accurate estimate of location (Fig. 1A left). If on the other hand, place cells have multiple place fields in the kinds of environments that must be navigated, then any combination of the independent location estimates from individual place cells will almost always provide an incorrect estimate of current location (Fig. 1A right), whereas the across-cell, ensemble pattern of current activity in the place cell population can accurately estimate location (Fig. 1B).

Although there have been reports of typical place cells with multiple firing fields in each hippocampus subregion, including CA3, what in our view is an important observation, has often been obscured by some other aspect of spatial coding that served as the focus of the study [9], [10]. This paper aims to draw attention to the fact that the fundamental spatial firing characteristic of place cells is to have multiple place fields, a possibility that was considered theoretically unlikely not so long ago [11]. We recorded place cells from each hippocampal subregion as a rat foraged for scattered food in a standard cylinder and a substantially larger box. Modifications in the recording conditions from our prior study eliminate potential procedural accounts for the finding of different place cell firing properties in small and large spaces.

## Materials and Methods

All experimental animal procedures have been previously published in detail, complied with NIH and institutional guidelines, and were approved by Downstate Medical Center's Institutional Animal Care and Use Committee.

### Subjects

Eight adult Long–Evans hooded male rats contributed data (Taconic Farms, NY). Electrophysiological data were analyzed from three animals and the other five animals were used to characterize computer-controlled foraging behavior. The electrophysiological methods were essentially similar to those in our previous study [6]. All differences are highlighted below.

### Apparatus

Two enclosures were used. The larger one was a 1.8 m×1.4 m box with medium gray walls. There were two holes in the center of each long wall, allowing a drinking spout to protrude into the box. The box was surrounded by black curtains, which could be drawn or closed. The box differed from the enclosure we used previously in that the long wall was 20% longer, there were no stairs to permit movements in 3-D, and the rat could not see far beyond the top of the box walls because of the surrounding curtains and/or room wall. The smaller enclosure was a 76-cm diameter cylinder with medium gray walls and a white card. The cylinder was placed in the center of the box. This cylinder is one of the most commonly used enclosures in place cell research [5] and was larger than the 68-cm diameter cylinder we used previously. The area enclosed by the box was 5.6 times greater than the cylinder's area.

### Position Tracking

We used a novel position tracking system that is commercially available (Bio-Signal Group, Brooklyn, NY). The system uses a pair of overhead cameras in a binocular package (Bumblebee, Point Grey Research, Richmond, B.C.) and binocular disparity to track locations in 3-D. Three-dimensional tracking allowed correction for two position-tracking errors that are inherent to all overhead video tracking systems and exaggerated in large environments. The first error arises due to optical distortion, which was corrected for in software after calibrating the visual field with a card of 10-cm gridlines. Binocular disparity was used to compute the tracked object's height, which was in turn used to correct the X and Y coordinates for the second error that arises due to parallax. Parallax occurs when we track the head of a rat in the horizontal plane and the animal changes its head elevation. The height displacement appears in a single camera as a horizontal displacement along the X and/or Y-axes away from the optical center of the camera. Parallax error grows as locations deviate away from the camera's optical center, which may be especially problematic in a large environment that permits 3-D movements.

We tracked the rat's location in 3-D at 40 ms resolution in a 76-cm diameter cylinder and a large rectangular box (1.8×1.4 m) with the binocular camera 2.5 m above the floor. The horizontal and vertical resolutions were 0.1 cm and 0.5 cm, respectively, after correcting for radial distortion from imperfect optics. We compared tracking in the horizontal plane with the 3-D system and a 2-D system created by ignoring information from the second camera. In the 2-D system, an 11 cm parallax error was caused at the box periphery by a vertical displacement of 24 cm, which is comparable to an adult rat rearing. Nonetheless, such errors did not significantly change place cell location-specific properties.

### Computer-controlled foraging behavior

A challenge to characterizing place cells in large spaces is the need for long recordings during which the animal continuously visits locations throughout all the accessible space. We used a foraging task that automatically trains rats to continuously visit all parts of an enclosure. The tracking system defined virtual, circular targets with diameters ∼40% of the short dimension of the enclosure, and only scattered food pellets when the rat was detected in the target. After pellet delivery, the target moved to another random location. In this way, the rat was reinforced for moving to different parts of the available space. Free access to drinking water was provided in the box in an effort to keep the rats foraging for the entire 60-min session. We took care to ensure that both enclosures were equally familiar to the rats in the electrophysiology experiments. The animals were exposed to the two enclosures in alternating sessions that lasted between 15 and 60 minutes each day.

### Electrophysiology

After 10 days of foraging training in the cylinder and box (without any other environmental manipulations), rats were anesthetized with Nembutal (50 mg/kg) and mounted in a stereotaxic frame. Eight independently movable tetrodes were implanted through a trephine hole in the skull above the dorsal hippocampus. The tetrodes were arranged in two rows within a custom microdrive assembly so that the array of tetrode tips occupied a 1.1 mm square. Relative to bregma, the center of the array was at AP 3.1, ML 2.8 for one rat and AP 3.8, ML 3 for two others. The electrode assembly was secured to the skull with bone screws and dental cement. After a week of recovery the rats were put back on food restriction and returned to the two enclosures for foraging. The tetrodes were subsequently advanced into the CA1, CA3, and DG regions until hippocampal action potentials were recorded. The signals were amplified 5000–10000 times, filtered between 300 Hz and 7 kHz, and digitized at 48 kHz using commercial hardware and software (dacqUSB, Axona Ltd., St. Albans, U.K.). Action potential waveforms were stored and analyzed offline. Single unit discrimination was done using custom software (Wclust, A.A. Fenton). Single units were studied only if they were sufficiently well discriminated using objective criteria based on *IsoI* estimates of isolation quality that were above 4 bits [6]. Once hippocampal discharge was identified, recordings were made in a 3-session cylinder–box–cylinder protocol. The cylinder sessions lasted 15 minutes and the box sessions were longer, lasting 60 minutes to obtain sufficient sampling of the accessible space.

### Electrophysiological analyses

Standard place cell analyses were performed as described previously [6]. Briefly, the overall, position independent firing rate of each cell was computed to estimate the cell's activity. Remapping was estimated as the degree to which activity differed between the cylinder and the box. The rate change ratio was computed as the absolute difference between the position-independent firing rates in the two environments divided by the maximum of the two rates. Cell-specific color-coded firing rate maps were computed as the total number of action potentials the cell emitted in each 2.8 cm square pixel divided by the total time the rat spent in the pixel. A place field was defined as any contiguous set of 9 or more pixels with greater that 0 AP/s firing rate that shared at least one side with another pixel in the field. Our initial work on CA1 used these same criteria [6], which are simpler and less restrictive than the firing field definition that was used in a recent related study [10]. The quality of spatial firing was estimated by the following standard parameters, spatial coherence [12] and spatial information content [13]. The values of these discharge measures were compared between the CA1, CA3, and DG cells using 1-way ANOVA followed by Scheffe's post-hoc tests as appropriate. We examined the spatial organization of multiple place fields in the large box by computing the spatial autocorrelation and characterizing the angle between the central peak and the two nearest neighbor peaks. We performed these analyses in our previous study [6], and repeated them here because they would detect the presence of spatial regularity if it existed, such as the hexagonal arrangement that is characteristic of entorhinal grid cell discharge [8].

### Histology

At the completion of useful recordings, the tetrode locations were verified by histological study. Electrolytic lesions were made prior to sacrifice by passing 20-µA anodal current for 20 sec from selected tetrodes. The rats were then transcardially perfused with PBS and then formalin. The formalin-fixed brain was removed from the skull and postfixed in the same solution for a week. Prior to cryostat sectioning, the brain was cryoprotected in 30% sucrose solution for two days. The brains were sectioned at 30–50 µm, stained with cresyl violet, and examined with a light microscope.

## Results

### Computer-controlled foraging behavior

**Figure 2:**
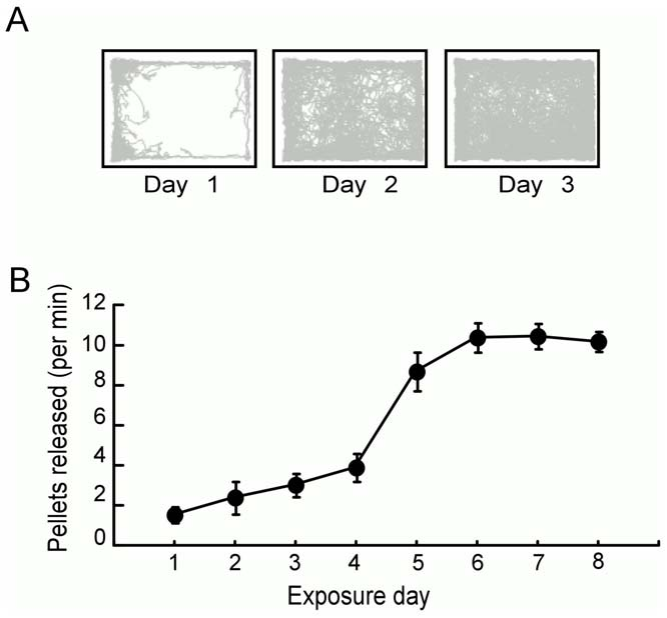
Computer-controlled foraging behavior. A) Examples of a rat's path through the box during its first 3 exposures. This rat learned to forage throughout the box rapidly. By the third 60-min session the rat was foraging throughout the box. B) Spatial exploration throughout the box improved rapidly with the computer-controlled foraging we implemented (F_7,28_ = 41.7, p = 10^−13^).

We trained five rats to forage for scattered food in the large box using the computer-controlled foraging task. The track of one rat during its first three exposures to the box is shown in Figure 2A and the average number of randomly located targets the rats entered to trigger the release of food is plotted as a function of session number in Figure 2B. The figures demonstrate that rats learned to forage throughout the box within a few sessions.

### CA1 place cells: multiple place fields in the box

**Figure 3:**
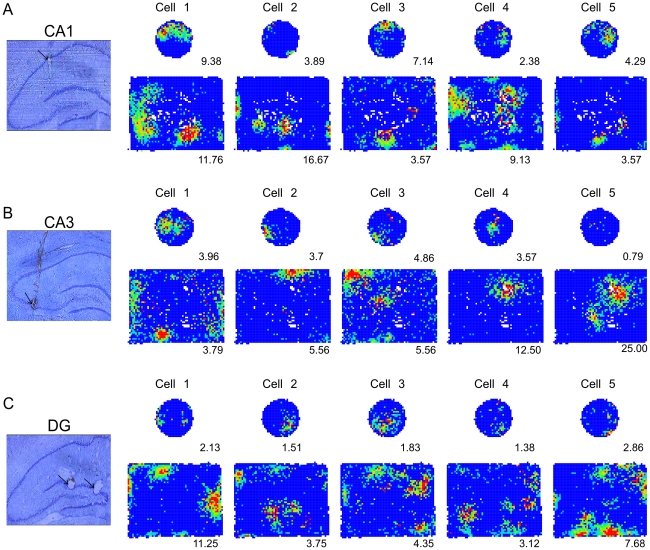
Examples of A) CA1, B) CA3, and C) DG spatial firing patterns in the cylinder and box. A histological section illustrating the recording location as well as five simultaneously-recorded place cells is given for each example. The number below each firing rate map is the lowest rate in the red color category.

**Figure 4:**
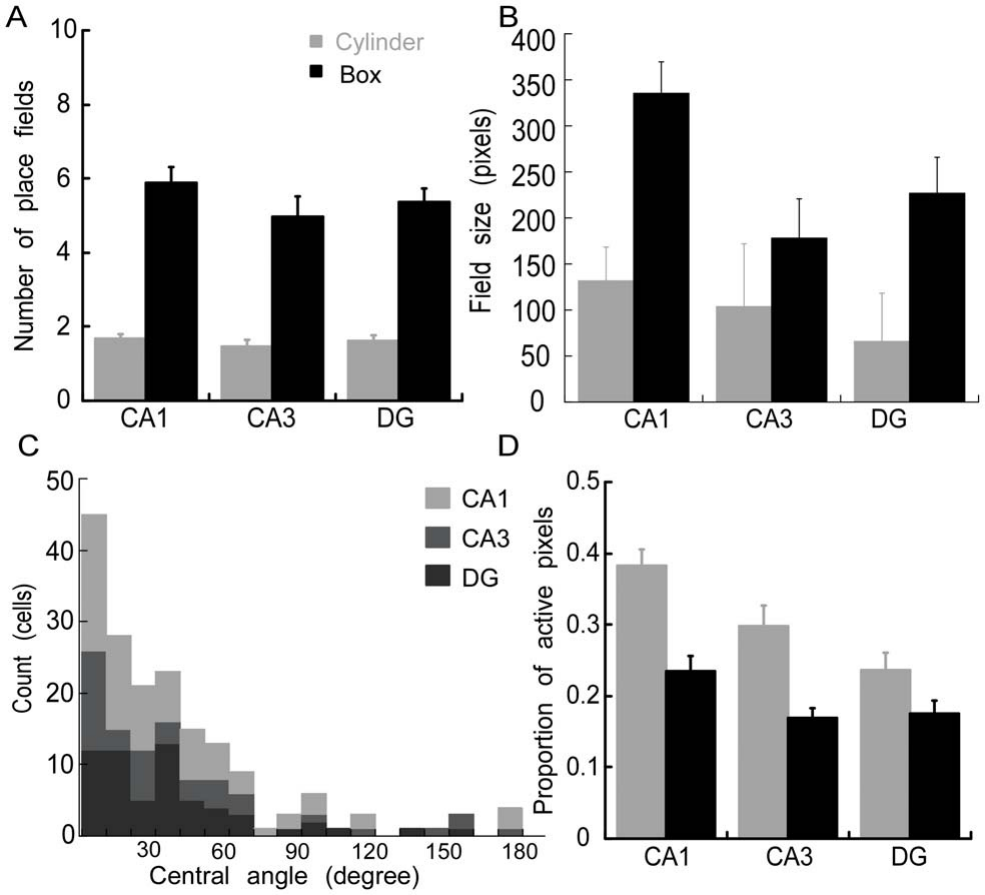
Comparison of CA1, CA3, and DG average place field properties in the cylinder and box. Place field A) number; B) size; and C) spatial organization, D) proportion of active pixels.

Recordings of 104 CA1 place cells in dorsal hippocampus (rat 1; 25 cells, rat 2; 27 cells, rat 3; 52 cells) showed that these cells have multiple place fields in the box (5.89±0.40 fields/cell; see Fig. 3A). Only a minority 17.9% (14/78) of the cells that were active in the box had a single place field. In contrast, the majority 60.7% (34/56) of cells that were active in the cylinder had a single place field in the smaller environment (test of proportions z = 7.73; p = 10^−15^). The single unit waveform isolation quality could not account for this difference (Table 1). The spatial arrangement of the place fields in the box was irregular, replicating prior observations (Fig. 4C).

**Table 1:** Summary of single unit isolation quality and its relationship to the number of place fields.

		*I_iso_(BG)*	*I_iso_(NN)*	Explained variance (r^2^) in number of place fields	
				*I_iso_(BG)*	*I_iso_(NN)*
CA1	Cylinder	6.52±0.15	8.72±0.31	0.0081	0.0036
	Box	6.76±0.18	8.67±0.31	**0.13**	0.0289
CA3	Cylinder	6.65±0.37	7.39±0.58	0.02	0.0081
	Box	7.14±0.20	8.39±0.39	0.07	0.01
DG	Cylinder	6.61±0.24	9.62±0.38	0.01	0.0121
	Box	7.04±0.14	8.97±0.37	**0.06**	0.0036

At most, only 13% of the variance in number of firing fields observed in the box is explained by single unit isolation quality. Note also that more cells are active in the box than the cylinder (Table 2; Fenton et al., 2008), making single unit isolation more difficult in the box. Significant Pearson correlations are in bold.

### CA3 place cells: multiple place fields in the box

The 52 place cells we recorded from the CA3 area of rat 2 also appeared to have multiple, irregularly-spaced place fields in the box (4.96±0.56 fields/cell; see Fig. 3B). The majority of these cells that were active in the cylinder 81.8% (18/22) had a single place field. In contrast, only 29% (12/41) of the cells that were active in the box had a single place field (test of proportions z = 8.18; p = 10^−15^). The single unit waveform isolation quality did not correlate with the number of place fields (Table 1).

### DG place cells: multiple place fields in the box

Most of the 58 place cells that were recorded from the region of the dentate gyrus of rat 3 had irregularly-spaced, multiple place fields in the box (5.35±0.38 fields/cell; Fig. 3C), as has been previously reported [2], [3]. While the majority of these cells, 63.9% (23/36) had a single place field in the cylinder just 8.2% (4/49) had a single field in the box (test of proportions z = 9.39; p<10^−16^). Errors in single unit waveform isolation could not account for the different numbers of place fields in the two environments (Table 1).

### Spatial firing properties compared across hippocampal subregions

We compared the number of place fields in the cylinder and box in each of the three hippocampal subregions (Fig. 4A). The average number of place fields was similar in the three subregions, which expressed over three times as many place fields in the box compared to the cylinder. The 2-way ANOVA comparing the effect of environment and subregion on the number of place fields per cell confirmed an effect of environment (F_1,341_ = 108.30.0; p = 10^−22^) but no effects of subregion (F_2,341_ = 0.83; p = 0.44) or the interaction (F_2,341_ = 0.41; p = 0.66).

Next we compared the properties of place fields in the two environments and across the three hippocampal subregions. We first examined the size of the firing fields. Since the tendency for multiple firing fields was much greater in the box than the cylinder, we compared the size of the largest place field for each cell (Fig. 4B). The effect of environment on field size was significant (F_1,341_ = 14.61, p = 0.0002) as was the effect of the hippocampus subregion (F_2,341_ = 3.20, p = 0.04). The interaction was not significant (F_2,341_ = 0.93, p = 0.39). Post-hoc tests confirmed that CA1 fields were larger than those in the other subregions (CA1>CA3 = DG). These differences in field size were obscured when all place fields were analyzed because this added a disproportionate number of small fields to the data set of fields from cells that were active in the large box. In this analysis, field size was not different in the cylinder and box (F_1,1316_ = 0.01 p = 0.93) and although there was a trend for CA1 place fields to be the largest, this was not statistically reliable (F_2,1316_ = 1.94; p = 0.14) and neither was the interaction of subregion and environment (F_2,1316_ = 0.31; p = 0.73). In any event, these data confirm prior observation that CA1 firing fields expand in larger environments [6], [14].

The arrangement of firing fields appeared to be irregular. In an effort to quantify the arrangement of firing fields, we set a threshold for defining a peak in the spatial autocorrelations. The threshold needed to be as low as 0.06 to define at least 3 peaks in 90% of the cells, which itself indicates spatial regularity was rather weak. Similar to what we previously reported for CA1 [6], the region-specific distributions of the angles between the three most prominent central peaks in the spatial autocorrelation tended to be significant and near 30°, according to the Rayleigh vectors (Fig. 4C; CA1: average (variance) = 32.8 (20.9°), r = 0.82; CA3: 32.7° (28.5°), r = 0.75; DG: 33.2° (14.9°), r = 0.87). We conclude that multiple firing fields in CA1, CA3 and the DG do not resemble hexagonal grids and are irregularly arranged.

We then computed the proportion of active pixels, locations in which the cells discharged at least one spike, which included the location of place fields as well as out-of-field spiking. The proportion of active pixels differed across both environments and subregion (Fig. 4D). The effect of environment was significant (F_1,341_ = 27.43; p = 10^−10^) because cells were active in a greater proportion of the cylinder than the box. The effect of subregion was also significant (F_2,368_ = 10.81; p = 10^−5^; post-hoc CA1>CA3 = DG), but the interaction was not (F_2,341_ = 1.53; p = 0.22).

**Figure 5:**
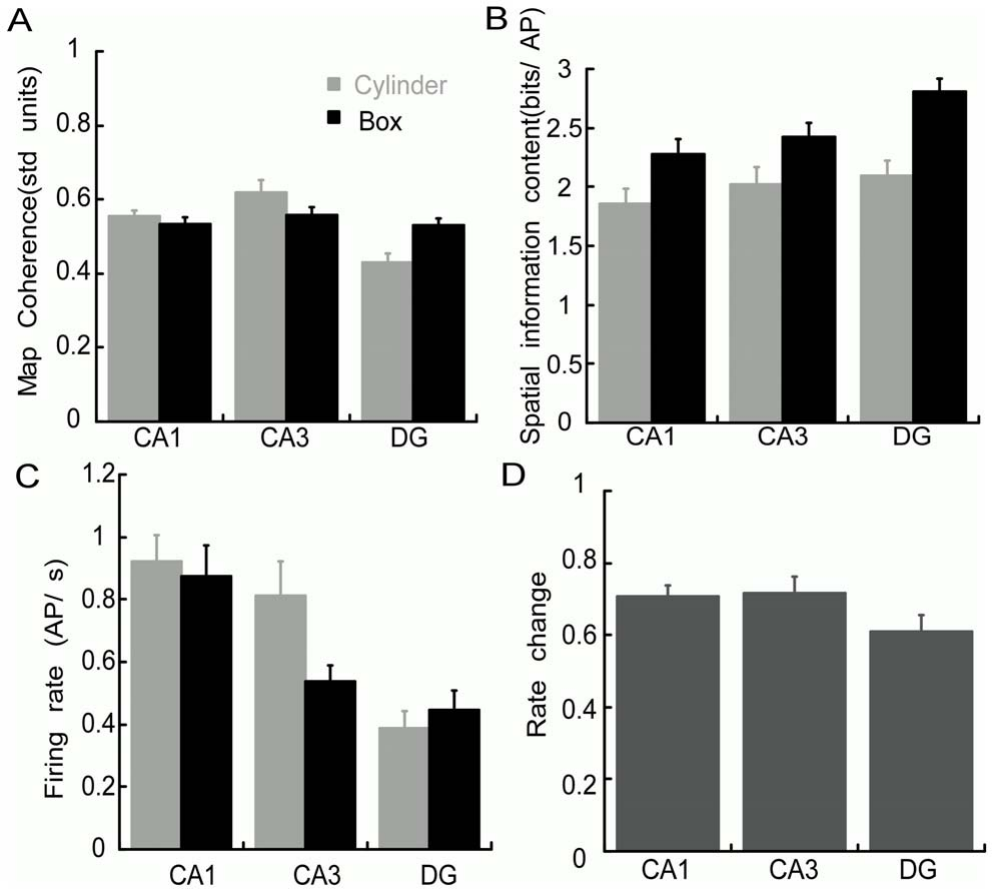
Comparison of CA1, CA3, and DG spatial discharge quality in the cylinder and box. Place field A) coherence; B) location-independent rate; C) information content; and D) rate change ratio (change in firing across environments).

We also examined the quality of spatial firing in the two environments and across the three hippocampal subregions. Spatial coherence (Fig. 5A) was similar in both environments (F_1,368_ = 0.14; p = 0.71) but different across the subregions (F_2,341_ = 9.48 p = 10^−5^), mainly because the coherence of DG cells was lower in the cylinder than the box (interaction: F_2,341_ = 6.21; p = 0.002 post-hoc CA1 = CA3>DG). The spatial information content (Fig. 5B) appeared to be similar in the cylinder across the subregions but higher in the box (F_1,341_ = 17.00; p = 10^−12^), with lower information content in CA1 cells and higher information content in CA3 and DG cells (F_2,341_ = 4.18; p = 0.016; post-hoc CA1<CA3 = DG). The interaction between environment and subregion was not significant (F_2,41_ = 0.63; p = 0.53). The position-independent firing rate (Fig. 5C) did not differ across environments (F_1,341_ = 0.99; p = 0.32), but was highest in the CA1 cells (F_2,341_ = 14.37 p = 10^−7^; post-hoc CA1>CA3 = DG). The interaction between environment and subregion was not significant (F_2,341_ = 1.15; p = 0.22).

### Remapping between the cylinder and box compared across hippocampal subregions

We also examined remapping between the two environments. Place cells that are active in one environment are not necessarily active in a second environment, because cells may turn on and off when the hippocampal place code remaps between environments [14]. We first identified the subset of principal cells that were recorded in both environments and expressed a place field in at least one environment. The change in activity between the two environments was first estimated by the rate change ratio, computed as the absolute difference in the position-independent activity in the two environments divided by the higher of the two rates. The rate change ratios (Fig. 5D) for each subregion were high, ∼0.7 indicating a substantial change across environments that did not differ across the subregions (F_2,196_ = 1.58; p = 0.21). We investigated further by estimating the active subset of cells as the proportion that was active (rate>0.1 AP/s) in a given environment (Table 2). In each subregion, the active subset was greater in the box than the cylinder (p's<0.001), suggesting that more place cells were recruited to represent the larger environment. This observation extends this finding from CA1 [6] to the CA3 and DG subregions.

**Table 2:** Remapping and the active subset across hippocampal subregions.

Subregion	Active in: cylinder	Active in: box	Active in: both	Cylinder vs Box test of proportions
CA1 (97)	57.73% (56)	68.04% (66)	25.77% (25)	z = 2.06, p = 10^−2^
CA3 (48)	45.83% (22)	77.08% (37)	22.92% (11)	z = 4.35, p = 10^−5^
DG (51)	58.82% (30)	86.27% (43)	45.09% (23)	z = 3.70, p = 10^−3^

In each subregion, a greater proportion of place cells were active in the box than the cylinder. Note that the proportion of DG cells that were active in both environments was higher than the proportions in the CA3 and CA1 areas (test of proportions z≥2.52, p<0.01). Only cells with an overall firing rate>0.1 AP/s in at least one environment were analyzed. The number of cells is given in parentheses.

**Figure 6:**
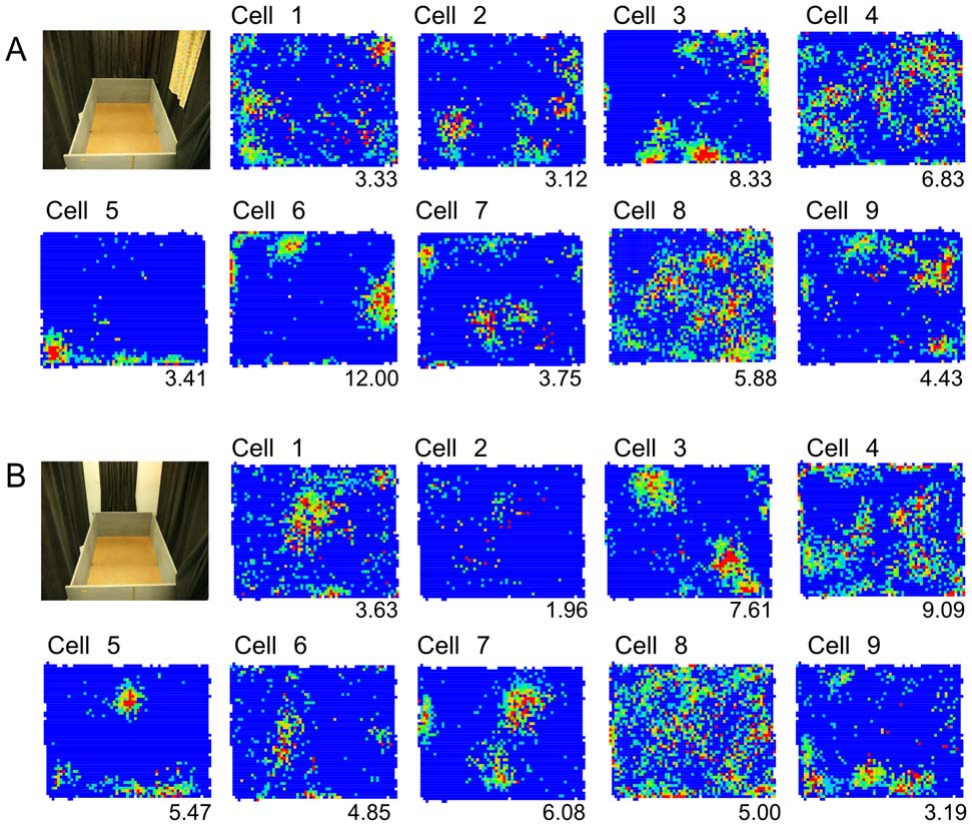
An example of remapping in an ensemble of DG cells. A) The standard and B) the altered visual environments. The blue-to-red color-coded firing rate maps are shown for a 9-cell ensemble of place cells that were recorded from the region of the dentate gyrus. The maps illustrate remapping between the two environments. The number below each map is the lowest rate in the red color category.

It has been reported that DG granule cells that are active in one environment are also likely to be active in another environment, substantially more that the cells in Ammon's horn [3], [15]. Consistent with these reports, the proportion of DG cells that were active in both the cylinder and box was greater than the proportions of cells in CA3 and CA1 that were active in both environments (p = 0.01 see Table 2). Nonetheless, in light of the idea that there is a constant active subset in the DG, it was surprising that the rate change ratios of the DG cells were as high as the values for CA1 and CA3. We considered the possibility that this discrepancy could be explained by the fact that our two environments differed substantially in size, whereas the size differential of the environments in prior work was far smaller. We made a single recording of 9 DG place cells in two similarly sized environments by changing the visual appearance of the surrounding (see Fig. 6). The rate change ratio for this experiment was 0.33±0.09, which was significantly smaller than the change we observed between the cylinder and the box (t_60_ = 2.51; p = 0.015). While our data are consistent with the idea that the DG active subset is relatively constant, they suggest that the active subset can change across environments that differ substantially in size.

## Discussion

### Summary

The main finding is that CA1, CA3, and DG place cells all characteristically discharged in multiple, irregularly-arranged place fields in the large box although the spatial discharge pattern of the vast majority of these cells simplified to a single place field in the 5.6 times smaller cylinder (Fig. 3). This replicated our prior report that the fundamental spatial firing pattern of CA1 place cells is to express multiple place fields. The results confirm prior observations of multiple firing fields in CA3 and DG place cells [3], [9], [10], [16] as well as other place cell spatial discharge characteristics. The prevalence of multiple place fields suggests that the hippocampus represents locations using an ensemble place code and is incompatible with notions of a dedicated hippocampal place code [6]. These observations strongly suggest there is a single, fundamental style of place coding throughout the subdivisions of hippocampus.

### Comparison to published work

The present work replicated and extends several published observations. First, the observation of multiple place fields in the present box cannot be explained by the rat's ability to move through the space in three dimensions, which was a possibility in our prior work [6] but not in the current study. As was reported for CA1, we could not detect any characteristic arrangement of firing fields from either the firing rate maps or their spatial autocorrelations. We replicated the observation that CA1 firing fields expand from small to large environments [6], which seems also to be the case for CA3 and DG place cells, although the effect was more modest. The observation that more principal cells are recruited to be place cells in a larger environment was also reproduced [6]. This extends the idea to DG and CA3 that the size of the active subset scales with environment size. The size of the hippocampal active subset appears not to be a constant.

CA3 place cells had multiple firing fields in the box. Indeed, multiple CA3 place fields was predicted in large spaces by a model in which the peak of a CA3 firing field arises from the linear combination of many active entorhinal cortical grid cell inputs and relatively few active dentate granule place cell inputs. In that model, the number of CA3 place fields was limited by both the sparse dentate excitation and a winner-take-all competition that was mediated by GABAergic inhibition [17] such that the opportunity to discharge in multiple places would only arise if a sufficiently large space was sampled.

On the surface, the observation of multiple CA3 place fields appears to be at odds with a report that only a minority of CA3 place cells have multiple firing fields on an 18-m long track. Subsequently, the same group reported multiple firing fields of CA3 place cells in a 4 m×4 m box [16] as did another group that recorded CA3 place cells on a linear track configured so that it resembled a figure-8, which allowed the rats to sample multiple directions [18]. Reasons for the discrepancies are unclear, but differences in environmental geometries may be factors. One reason for the importance of environmental geometry could be the reduction of spatial sampling to a single direction on the 18-m track. This difference would be significant, compared to an open field, if directionally tuned cells make a strong contribution to discharging place cells such that location-specific excitation of a place cell will remain sub-threshold without a directional input. This is indeed the case, as CA3 cells on linear tracks, including the 18-m one, only tend to discharge in a firing field when the rat runs through the field location in a single direction. This strong modulation of firing by direction suggests the importance of head direction in discharging CA3 place cells because environmental stimuli as well as the spatial firing of entorhinal layer II grid cell inputs to CA3 are not modulated by direction [19]. It was also reported that CA1 place cells have multiple firing fields in enlarged open environments and multiple fields were more likely in distal CA1 than proximal CA1 [10]. Although proximal-distal differences in CA3 place cell discharge properties have not been described, differences may be expected on the basis of the topography of CA3-CA1 connectivity, which may also contribute to the discrepancies with single CA3 firing fields on the 18-m track.

We also observed that most DG place cells had multiple firing fields in the box, which is consistent with reports that DG cells have multiple firing fields [2], [3], [15]. We observed that DG cells changed firing rates between the cylinder and box as much as cells in CA1 and CA3, which remapped. While such changes are at odds with reports that individual DG cells characteristically remain active across multiple environments [3], [15], we found that the proportion of DG cells that were active in both environments was higher than the corresponding proportions in the CA1 and CA3 regions, and furthermore, that the DG active subset was more constant when recordings were made in two similarly-sized environments, observations that are consistent with the published work. We emphasize that the aim of the present study was not to investigate remapping, and all the DG recordings in which remapping could be evaluated were from a single rat, making the generality of these observations uncertain. Furthermore, we did not use perforant path stimulation or otherwise verify that our DG recordings were from granule cells, leaving open the possibility that there are at least two different populations of DG place cells.

### Ensemble coding throughout the hippocampus

Spatially tuned hippocampal neurons characteristically express multiple place fields in sufficiently large environments. While these observations are compatible with the popular notion that place cells characteristically discharge in a single location in the small, laboratory environments that are in standard use, the observations are incompatible with thinking that location is accurately represented by any average of the location estimates of individual place cells (Fig. 1). On the contrary, multiple place field discharge patterns are entirely compatible with ensemble coding schemes in which the across-cell, ensemble discharge at each location is unique. Location-specific ensemble discharge is likely to be maintained across large expanses at every stage of the perforant path through the hippocampus because of the multiple, irregularly-arranged place fields of these cells. It appears that an ensemble place code must be in operation at every stage of the perforant path.
